# Decrease of UPR- and ERAD-related proteins in *Pichia pastoris* during methanol-induced secretory insulin precursor production in controlled fed-batch cultures

**DOI:** 10.1186/1475-2859-13-23

**Published:** 2014-02-13

**Authors:** Ana Letícia Vanz, Manfred Nimtz, Ursula Rinas

**Affiliations:** 1 Leibniz University of Hannover, Technical Chemistry – Life Science, Callinstr, 5, 30167 Hannover, Germany; 2 Helmholtz Centre for Infection Research, Inhoffenstrasse 7, Braunschweig 38124, Germany

**Keywords:** Endoplasmic reticulum associated degradation (ERAD), Insulin precursor, Methanol metabolism, *Pichia pastoris*, Proteome, Two-dimensional gel electrophoresis, Unfolded protein response (UPR)

## Abstract

**Background:**

*Pichia pastoris* is a popular yeast preferably employed for secretory protein production. Secretion is not always efficient and endoplasmic retention of proteins with aberrant folding properties, or when produced at exaggerated rates, can occur. In these cases production usually leads to an unfolded protein response (UPR) and the induction of the endoplasmic reticulum associated degradation (ERAD). *P. pastoris* is nowadays also an established host for secretory insulin precursor (IP) production, though little is known about the impact of IP production on the host cell physiology, in particular under industrially relevant production conditions. Here, we evaluate the cellular response to *aox1* promoter-controlled, secretory IP production in controlled fed-batch processes using a proteome profiling approach.

**Results:**

Cells were first grown in a batch procedure using a defined medium with a high glycerol concentration. After glycerol depletion IP production was initiated by methanol addition which was kept constant through continuous methanol feeding. The most prominent changes of the intracellular proteome after the onset of methanol feeding were related to the enzymes of central carbon metabolism. In particular, the enzymes of the methanol dissimilatory pathway - virtually absent in the glycerol batch phase - dominated the proteome during the methanol fed-batch phase. Unexpectedly, a strong decrease of UPR and ERAD related proteins was also observed during methanol-induced IP production. Compared to non-producing control strains grown under identical conditions the UPR down-regulation was less pronounced indicating that IP production elicits a detectable but non prominent UPR response which is repressed by the general culture condition-dependent UPR down-regulation after the shift from glycerol to methanol.

**Conclusions:**

The passage of IP through the secretory pathway using an optimized IP vector and growing the strain at fed-batch conditions with a high initial glycerol concentration does not impose a significant burden on the secretory machinery even under conditions leading to an extracellular accumulation of ~ 3 g L^-1^ IP. The glycerol batch pre-induction culture conditions are associated with a high constitutive - recombinant protein production independent - induction of the UPR and ERAD pathways probably preconditioning the cells for effective IP secretion in the methanol fed-batch phase.

## Background

The methylotrophic yeast *Pichia pastoris* is a well-established eukaryotic host for the production of heterologous proteins preferentially secreted into the medium to simplify further down-stream procedures [1,2]. Secretory protein production usually requires the presence of a signal sequence at the N-terminus of the foreign protein to target it to the secretory pathway, namely allowing transfer of the protein into the endoplasmic reticulum (ER), passage through the Golgi apparatus, and, finally, vesicular transport to the extracellular environment. However, not all recombinant proteins are efficiently secreted and ER retention during high-level production can be a problem. In particular, aberrant folding properties of the target protein and/or high level production can lead to the accumulation of unfolded or even aggregated proteins in the ER [3-6] which can initiate the unfolded protein response (UPR) [5-10] and ER-associated degradation (ERAD) [5-7].

Simplified, overloading of the secretory pathway is sensed in the ER by binding of KAR2 (or BiP) to folding intermediates or misfolded proteins which outcompete the binding of KAR2 to the ER luminal domain of IRE1 [11-13]. The release of KAR2 from the luminal IRE1 domain leads to conformational changes and autophosphorylation and subsequent activation of the cytosolic IRE1 endoribonuclease domain. This leads to splicing of the IRE1 substrate, *HAC1* mRNA, being transformed into the activated form encoding the transcriptional activator of UPR responsive genes, Hac1p [11-13].

Many UPR responsive genes encode ER resident chaperones and foldases, including the most prominent ER chaperone, KAR2, and the major ER disulfide isomerise, PDI. In addition to acting as the sensor protein for the presence of un-/misfolded proteins, KAR2 is also involved in chaperoning protein folding thereby relieving from protein (mis) folding associated stress in the ER [14]. PDI is responsible for disulfide exchange reactions in the ER helping to rearrange incorrect disulfide pairings [15]. Both proteins as well as Hac1(p) have been co-overproduced in *P. pastoris* for enhanced secretion of target proteins with mostly unpredictable and varied success [3,12,16-20].

The induction of the UPR response in *P. pastoris* through recombinant protein production was mainly studied using a transcriptome based approach [5,6,8-10,21]. In all these cases transcript or transcriptome analysis revealed enhanced expression of UPR-related genes during secretory recombinant protein overproduction [5,8-10] or ERAD-related genes in case of a high propensity of the recombinant protein to misfolding an/or retention in the ER [5,6]. Moreover, UPR induction was also detected by increasing levels of KAR2 (protein) upon secretory recombinant protein overproduction [22,23]. Recently, also more comprehensive proteomic studies were carried out to monitor the induction of the UPR and other stress responses during recombinant protein production in *P. pastoris*[7,19]. These studies revealed increased levels of UPR related proteins upon methanol induced production of secretory xylanase [19] and ER residing Hepatitis B surface antigen [7]. However, only the production of the Hepatitis B surface antigen, a protein retained in the ER [4], also led to an ERAD response apparent through the strong increase of two cytosolic chaperones and members of the AAA ATPase superfamily which are participating in ERAD [7]. No ERAD response was observed during secretory xylanase production [19]. UPR and ERAD are coordinated responses in yeast as has been shown in more detail for *Saccharomyces cerevisiae*[24], however, a strong ERAD response might only be necessary if the ER cannot get cleared by a regular and coordinated passage of the target protein through the entire secretory pathway.

In this study we have analyzed the cellular response towards methanol induced secretory insulin precursor (IP) production with special attention to the occurrence of proteins related to the UPR and ERAD pathways. Surprisingly, our analysis revealed a decrease of UPR and ERAD related proteins in response to secretory IP production under industrially relevant production conditions.

## Results and discussion

A proteome profiling approach was chosen to evaluate the cellular response of *P. pastoris* towards secretory IP production under industrially relevant production conditions. The recombinant strain was first grown to high-cell density in a batch procedure using a defined medium with low salt and high glycerol concentrations [25]. After depletion of glycerol, secretory IP production was induced by methanol addition to a final concentration of 2 g L^-1^ which were kept constant by continuous methanol feeding leading to final extracellular IP concentrations of ~ 3 gram per liter of culture broth [25] with less than 10% IP remaining intracellular (data not shown). The details of the original cultivation data are given elsewhere [25] and the list of all identified proteins is shown in the Additional file 1: Table S1.

### General proteomic response towards methanol-induced secretory IP production in controlled fed-batch culture

**Figure 1:**
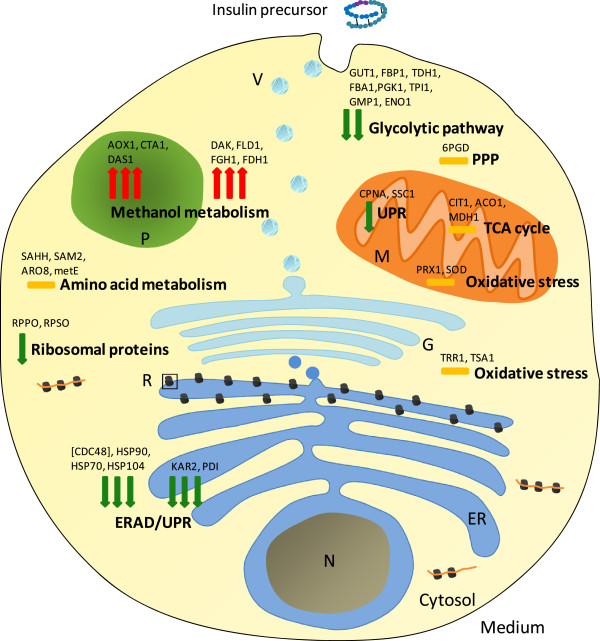
**Global view on the intracellular proteome profile change in *P. pastoris *X-33 during secretory insulin precursor production (after shift from glycerol batch to methanol fed-batch).** The red arrows (↑) indicate an increasing and the green arrows (↓) a decreasing amount of functional proteins in the methanol fed-batch phase. An orange dash (−) indicates no significant change. One arrow indicates small (log2 change 0.6-1), two arrows strong (log2 change 1–4) and three arrows very strong changes (log2 change > 4). The arrows correspond to the average of log2 fold changes of proteins from each functional group. The position of the arrows is according to the proteins location in the cell. Only the most prominent proteins from each functional group are indicated. The complete list of all identified proteins and the corresponding values of log2 changes are given in the Additional file 1: Tables S1 and S2, respectively. Abbreviations: TCA, tricarboxylic acid cycle; PPP, pentose phosphate pathway; ERAD, endoplasmic reticulum associated degradation; UPR, unfolded protein response; P, peroxisome; R, ribosome; ER, endoplasmic reticulum; V, vesicle; M, mitochondria; N, nucleus; G, Golgi complex. Protein/gene abbreviations are given in the Additional file 1: Table S1.

**Figure 2:**
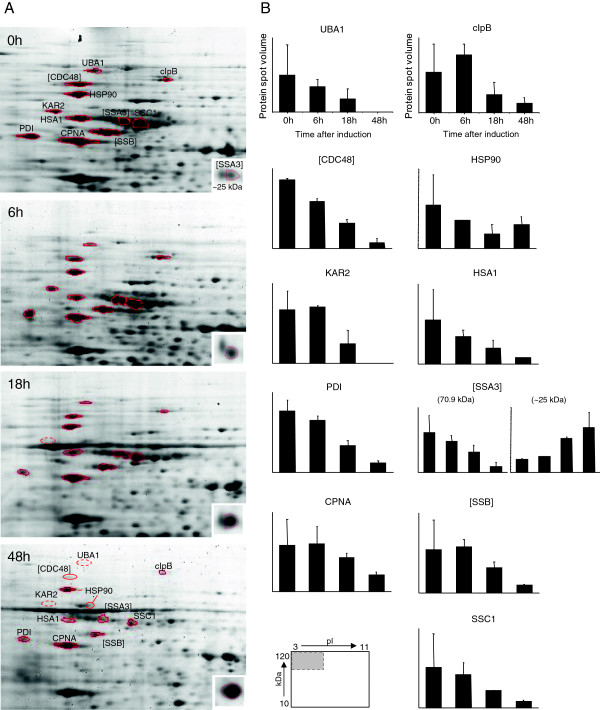
**Change of the intracellular proteome of *P. pastoris *X-33 in response to secretory insulin precursor production with special attention to ERAD and UPR related proteins. (A)** Sections of 2D gels containing most of ERAD and UPR related proteins are shown: samples taken at 0, 6, 18 and 48 h after the onset of the methanol fed-batch phase. **(B)** Abundance changes of ERAD and UPR related proteins are given in relative units corresponding to an average of the resulting values from duplicate gels. The protein (spot) related to the fragment of SSA3 ~25 kDa is shown in the small box. The small map indicates the position of the 2D sections in the entire 2D gel. Two representative 2D gels from cell samples taken at the end of the glycerol batch phase and during methanol-induced secretory IP production indicating the position of all identified proteins are given in the Additional file 1: Figure S1. Time course 2D data from a replicate cultivation are given in the Additional file 1: Figure S2.

**Figure 3:**
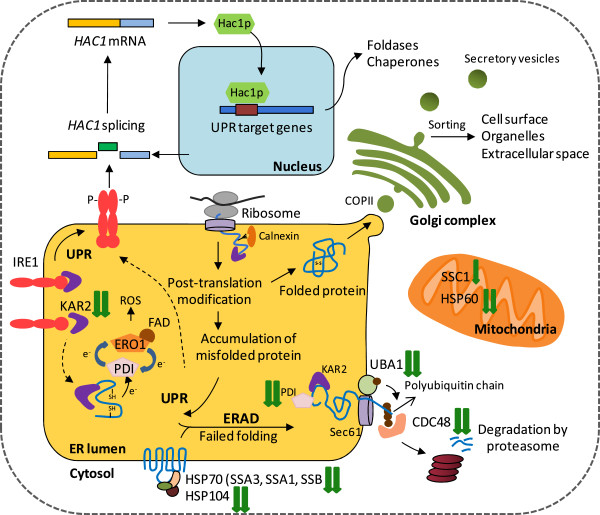
**Global view on the ERAD and UPR responses of *P. pastoris *X-33 during secretory insulin precursor production (after shift from glycerol batch to methanol fed-batch).** (Identified) proteins related to UPR and ERAD responses, their function, cellular location and abundance changes during secretory insulin precursor production are indicated. The green arrows (↓) indicate the identified proteins with decreasing abundance, with one arrow indicating small (log2 change 0.6-1) and two arrows strong decreases in abundance (log2 change 1–4).

Glycerol and methanol were the sole carbon sources during the growth and production phases, respectively. Accordingly, enzymes involved in carbon source metabolism showed the most prominent changes during the production phase (Figure 1, Additional file 1: Figure S1 and Additional file 1: Table S2). In particular, the enzymes involved in methanol dissimilation increased most drastically during the methanol fed-batch phase in agreement with previous observations made during methanol-induced production of the Hepatitis B surface antigen with *P. pastoris*[7]. Also, the enzymes of the methanol dissimilation pathway (AOX1, CTA1, FLD1, FGH1 and FDH1) increased more rapidly compared to those enzymes involved in methanol assimilation (DAS1, DAK) after the onset of methanol feeding (Additional file 1: Table S2) also in agreement with previous findings [7]. This finding is also in accordance with the observed growth arrest in the early phase of the methanol fed-batch phase [25,26] reflecting the more urgent need of cells for methanol catabolizing enzymes to generate sufficient energy to reconstruct the proteome prior to synthesizing the enzymes for incorporation of methanol into product/biomass. The enzymes of the other common central metabolic pathways either declined in abundance (e.g. glycolytic pathway) or did not show significant changes (e.g. TCA cycle, pentose phosphate pathway) in the methanol fed-batch phase (Figure 1, Additional file 1: Figure S1 and Additional file 1: Table S2). Also, cellular proteins belonging to other functional categories (e.g. oxidative stress response, amino acid metabolism) did not show a clear trend concerning their changes in abundance while ribosomal proteins revealed a slight decrease in abundance presumably as a result of the lower growth rate in the methanol fed-batch phase (Figure 1, Additional file 1: Figure S1 and Additional file 1: Table S2). The most unexpected finding concerning the reconstruction of the yeast proteome after the shift from growth on glycerol to methanol-induced secretory IP production was related to proteins involved in the UPR and ERAD pathways. These proteins did not increase during the methanol fed-batch phase as anticipated but revealed a drastic decrease in abundance (Figures 1, 2, 3, Additional file 1: Figures S1 and S2 and Additional file 1: Table S2).

### UPR and ERAD responses during secretory IP production in controlled fed-batch culture

Despite the high-level secretory production of a foreign protein, UPR and ERAD related proteins strongly decreased in the IP producing strain during the methanol fed-batch phase (Figures 1, 2, 3, Additional file 1: Figures S1 and S2, and Additional file 1: Table S2). A significant decline of many UPR-related proteins became already apparent 18 hours after the onset of methanol feeding (Figure 2). This included the most prominent chaperone of the ER, KAR2 (also known as BiP) which decreased to almost undetectable levels (Figure 2 and Additional file 1: Figure S2). In addition to the typical ER resident chaperones and foldases (e.g. KAR2, PDI) also cytosolic and mitochondrial chaperones decreased in abundance during the methanol fed-batch phase (Figures 2, 3, Additional file 1: Figures S1 and S2, and Additional file 1: Table S2). For example, the identified cytosolic chaperones with decreasing abundance, e.g. members of the HSP70 family (SSA1, SSA3 and SSB) are encoded by UPR-responsive genes which show increased expression in Hac1p overproducing strains [21]. Moreover, two cytosolic chaperones and members of the AAA ATPase superfamily (ClpB = HSP104 and the AAA ATPase PAS_FragD_0026 = CDC48) also decreased strongly in abundance during the methanol fed-batch phase (Figures 2, 3, Additional file 1: Figures S1 and S2, Additional file 1: Table S2). Both proteins are members of the ERAD pathway which ultimately target misfolded proteins from the ER to cytosolic proteasomal degradation [7]. For example, the AAA ATPase CDC48 is a ubiquitin-binding protein engaged in the delivery of multi-ubiquitinated proteins to the proteasome for final degradation [27,28]. In this line, the ubiquitin activating enzyme UBA1, which catalyses the first step in ubiquitination [29] also decreased in abundance after the shift from the glycerol batch to the methanol fed-batch phase (Figures 2, 3, Additional file 1: Figures S1 and S2, and Additional file 1: Table S2). The mitochondrial chaperones CPNA (HSP60 family) and SSC1 (HSP70 family) which also revealed a decreasing abundance in the methanol fed-batch phase are not directly involved in recombinant (secretory) protein folding and degradation, but are classical UPR targets induced by HAC1 overexpression or dithiotreitol addition [21]. The observed decrease of UPR and ERAD related proteins in the methanol fed-batch phase strongly suggests that other non-recombinant protein related effects might be responsible for their observed decline during IP production. Moreover, the low increase of biomass during the production phase [25] also suggests that the restructuring of the intracellular proteome after growth on glycerol to methanol induced IP production results from de- and reconstruction processes and not simply from dilution and de novo synthesis.

### UPR response in host (control) and IP producing strains at different methanol concentrations in shake flask culture

**Figure 4:**
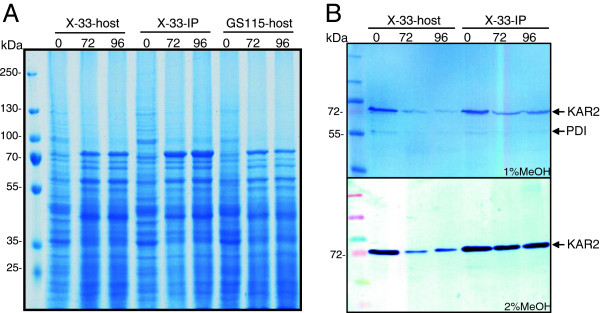
**UPR response in secretory insulin precursor producing *P. pastoris *X-33-IP and non-producing host strains X-33 and GS115.** Cells were grown on glycerol in shake flask cultures (same glycerol concentration as in bioreactor cultures) and resuspended in medium containing methanol. Samples were taken at the end of the glycerol phase directly before induction with methanol (0) and at 72 and 96 h after induction with 1% or 2% methanol. **(A)** Crude cell lysates from 1% methanol cultures of the secretory insulin precursor producing *P. pastoris* X-33 (X-33-IP), and the non-producing host strains *P. pastoris* X-33 (X-33-host) and GS115 (GS115-host) were analyzed by SDS-PAGE. **(B)** Crude cell lysates from 1% and 2% methanol cultures of the secretory insulin precursor producing *P. pastoris* X-33 (X-33-IP) and the non-producing host *P. pastoris* X-33 (X-33-host) were probed for proteins containing the endoplasmic reticulum retention signal peptide HDEL (e.g. KAR2, 74 kDa and PDI, 58 kDa) by Western Blot analysis.

To discriminate between culture condition and recombinant protein synthesis dependent effects on the UPR response during methanol-induced secretory IP production, cultivations under identical conditions were performed using the IP producing and non-transformed host strains. Cells were first grown in shake flasks on glycerol at concentrations also employed in bioreactor cultivations and subsequently subjected to methanol containing medium. The induction of the UPR response was assessed by probing for proteins containing the HDEL ER retention peptide using an anti-HDEL antibody. Interestingly, the amount of the most prominent UPR protein KAR2 (or BiP) was highest prior to the addition of methanol and declined after ongoing incubation in methanol containing medium in the IP producing but also in the non-transformed host strain (Figure 4). However, the decline of KAR2 in response to methanol addition was less prominent in the IP-producing strain compared to the non-producing host suggesting that production of IP elicits a detectable but non prominent UPR response which is repressed by the general culture condition-dependent UPR down-regulation after the shift from glycerol to methanol. However, at very high methanol concentrations leading to stronger *aox1*-dependent gene expression, respectively elevated IP synthesis, the culture condition dependent down-regulation of the UPR response is superimposed by the UPR induction through enhanced IP synthesis leading to non-declining levels of the UPR responsive proteins (Figure 4).

## Conclusions

During methanol-induced secretory IP production a general decrease of UPR and ERAD related proteins occurred within *P. pastoris* at the culture conditions employed. Compared to the non-producing host strain the UPR down-regulation was less pronounced indicating that production of IP elicits a detectable but non prominent UPR response which is repressed by a general culture condition dependent UPR down-regulation after the shift from glycerol to methanol. Appearance and removal of misfolded proteins are inherent incidents during normal growth [24] which might be differently regulated at different environmental conditions. Previous findings indicated that the extend of the UPR in yeast is also connected to the nutritional state of the cell [11] and, as has been shown for *P. pastoris*, also to other environmental conditions such as osmolarity [30] and temperature [31]. Environmental factors are presumably responsible for the high level of UPR and ERAD related proteins in the batch phase in which high concentrations of glycerol were employed. Moreover, there are also - though not yet conclusive - indications that *P. pastoris* may exhibit in general a stronger constitutive or basal induction of the UPR as other yeast independent of the production of aberrant proteins [6,12,13]. Our findings also indicate that IP is a quite stable protein as it has been shown that the native-state stability of a secreted protein is inversely correlated to its UPR and ERAD inducing effect [5]. Thus, the properties of IP, e.g. its stability as well as the moderate induction conditions do not provoke significant folding stress during IP production in the controlled fed-batch process. Above all, the high levels of UPR related proteins prior to induction probably precondition the cells for effective IP secretion in the methanol induction phase and this “more constitutive” UPR induction might be responsible for the superior secretion properties of *P. pastoris*.

## Materials and methods

### Strains and growth conditions

#### Strains

The *P. pastoris* host strains X-33 and GS115 were from Invitrogen (Carlsbad, CA, USA). Details of the construction of the recombinant *P. pastoris* strain X-33 carrying a codon-optimized copy of a synthetic IP gene for secretory IP production under the control of the *aox1* promoter (Mut^+^) and usage of the α-factor secretory signal are given elsewhere [25].

#### Bioreactor fed-batch cultivations

Growth and insulin precursor production under industrially relevant conditions using a defined medium were essentially carried out as described before [25]. Cells were first grown in a batch procedure using glycerol as sole carbon source with an initial glycerol concentration of 95 g L^-1^[25,26]. After depletion of glycerol, insulin precursor production was induced by a pulsed methanol addition and subsequent methanol feeding to maintain the methanol concentration at 2 g L^-1^[25]. Initially, the methanol concentration was increased in a step-wise manner to the final concentration of 2 g L^-1^ but in follow-up cultivations the methanol concentrations were immediately increased to 2 g L^-1^ without detectable effects on the final product yield and cell responses (see also Additional file 1: Figure S2).

#### Shake flask cultivations

500 mL baffled shake flasks containing 100 mL basal medium (20 g L^-1^ glycerol, 13.4 g L^-1^ yeast nitrogen base without amino acids, 400 μg L^-1^ biotin in ddH_2_O) were inoculated from glycerol stocks. The cultures were grown for approximately 36 h at 30°C and 250 rpm to an OD_600_ 8–10 and used to inoculate the next preculture (1% inoculum, 100 mL basal medium). This preculture was grown for approximately 20 h (OD_600_ 3–5) and taken as an inoculum for the main culture (10% inoculum, 450 mL defined medium, 2 L baffled shake flasks). The defined medium was identical to the medium employed for the glycerol batch phase in bioreactor cultures [25,26]. The main cultures were grown for 30–40 h at 30°C and 150 rpm, the cells collected by centrifugation, washed with sterile PBS and resuspended in defined medium without glycerol to OD_600_ 100. Recombinant protein production was induced by the addition of 1% or 2% methanol (every 12 h repeated).

### Sample preparation for two-dimensional gel electrophoresis, two-dimensional gel electrophoresis, and protein spot identification and quantification

All procedures were carried out essentially as described previously [7]. After harvesting by centrifugation, cell pellets were immediately flash-frozen in liquid nitrogen and kept at −80°C before further treatments.

#### Sample preparation

Cell pellets were washed with ice-cold phosphate-buffered saline (PBS), resuspended in 1 mL cell lysis buffer (7 mol L^-1^ urea, 2 mol L^-1^ thiourea, 4% (w/v) Triton X-100, 30 mmol L^-1^ Tris, pH 8.5) with the OD_600_ adjusted to OD 50 and combined with 500 μL of glass beads (0.5 mm, Sartorius, Germany). For cell disruption, samples were treated twice in a Thermo Savant Fastprep FP120 homogenizer (speed 6.00 m/s for 30 s; cooling interval of 30 s between treatments). Following, cell debris was removed by centrifugation at 13000 rpm and 4°C for 5 min and proteins in the supernatant precipitated using chloroform and methanol. The protein pellets were air-dried and dissolved in 500 μL of resolubilization solution (9 mol L^-1^ urea, 2 mol L^-1^ thiourea, 4% CHAPS, 2 mg mL^-1^ Tris, 0.2% SDS, 0.002% bromophenol blue). To this solution, 7.5 μL IPG buffer (Amersham Biosciences, UK) and 7.5 μL 1 mol L^-1^ dithiothreitol were added and the resolubilized proteins stored at -80°C until further analysis.

#### Two-dimensional gel electrophoresis

The first-dimension of isoelectric focussing (IEF) was carried out using the IPGphor™ Isoelectric Focussing System (Amersham Biosciences, UK) at 20°C with a current of 30 μA per strip. 400 μg of each protein sample were loaded onto Immobiline DryStrip gels of pH 3–10 NL (Amersham Biosciences, UK) by in-gel rehydration. IEF was performed with the following setting: 0 V × 35 h, 50 V × 4 h, gradient from 100 V to 300 V within 4 h, gradient from 300 V to 1000 V within 3 h, gradient from 1000 V to 3500 V within 4 h, gradient from 3500 V to 5000 V within 3 h, 5000 V × 3 h, gradient from 5000 V to 8000 V within 3 h, then 8000 V × 10 h. The second-dimension was carried out using 12% SDS-PAGE gels and the vertical separation unit Hoefer™ System (Amersham Biosciences) at 10°C in constant working voltage mode as follows: 40 V for 2 h and then 100 V overnight. Subsequently, gels were stained using colloidal Coomassie Brilliant Blue G-250 according to the “Blue silver” protocol [32]. The gels were then scanned (Epson Perfection V750 Pro, Epson, Germany) at 300 dpi resolution to acquire the gel images.

#### In-gel trypsin digestion and peptide extraction

Protein spots were excised manually from the stained gels, washed several times with 200 μL water, dehydrated in 200 μl acetonitrile, and dried in a vacuum concentrator (Eppendorf® Vacufuge Concentrator 5301, Eppendorf AG, Hamburg). The gel pieces were treated with 100 mmol L^-1^ ammonium bicarbonate, containing 20 mmol L^-1^ DTT at 56°C for 30 min and then with 100 mmol L^-1^ ammonium bicarbonate containing 55 mmol L^-1^ iodoacetamide in the dark at room temperature for 30 min. Acetonitrile was added in between the treatments to dehydrate the gel pieces. Finally, the gel pieces were washed twice with 100 mmol L^-1^ ammonium bicarbonate, dehydrated with acetonitrile and dried in the vacuum concentrator. In-gel digestion was carried out by incubation with 2 ng μL^-1^ trypsin (sequencing grade modified, Promega Corp., USA) in 50 mmol L^-1^ ammonium bicarbonate at 37°C overnight. Obtained peptides were extracted, washed with a buffer for desalting (10 mmol L^-1^ ammonium phosphate, monobasic in 0.1% trifluoroacetic acid (TFA) and then loaded to a Prespotted Anchor Chip (Bruker Daltonics GmbH, Germany) targeted for MALDI-TOF analysis. The molecular masses of the tryptic peptides were determined on a Bruker Ultraflex time-of-flight mass spectrometer (Bruker Daltonics GmbH, Germany).

#### Protein identification and quantification

Peptide mass fingerprints obtained by the MALDI-TOF MS were processed using FlexAnalysis 2.0 (Bruker Daltonics GmbH, Germany) and used to search the NCBInr database by using Mascot 2.10 software (http://www.matrixscience.com). The parameters used for searching were as follows: taxonomy: other Fungi, tryptic digestion, modifications were allowed for carbamidomethylation of cysteine (fixed modification) and methionine oxidation (variable modification), one missed cleavage site was allowed, all peptides monoisotopic, peptide tolerance at 100 ppm. Mascot scores (probability based MOWSE scores) and expect values were generated using the Mascot search program. All proteins with a Mowse score greater than 71 were regarded as significant (p < 0.05). For most peptide mass fingerprints, a single significant hit (*P* < 0.05) with a probability-based Mowse score greater than 71 was obtained. In rare cases the Mowse score was below 71, which indicated that the protein was not identified with reliability above the level of significance. These protein spots were excluded from the results unless the identification was confirmed by MS/MS. Image analysis from the scanned gels, namely protein spot detection, matching and quantification were performed using Proteomweaver™ 3.0 (Definiens AG, Germany). For each sample, 2D gels were made in triplicate. And the best two gels were analyzed. The spot volumes were computed and normalized for each spot on each gel in relation to the total spot volume of each 2D gel. To obtain comparable data, spot intensities were normalized, using the log2 ratio of induced samples *versus* uninduced samples. Log2 fold changes above 0.6 (equivalent to a 1.5 fold change) were considered significant. The gene name used is this study is according to *P. pastoris* strain GS115 (http://www.uniprot.org/). If no gene name is given for this strain the gene name is according to *P. pastoris* strains (ATCC 76273/CBS 7435/CECT 11047/NRRL Y-11430/Wegner 21–1) or *P. pastoris* (yeast) in case of 100% sequence identity (http://www.uniprot.org/).

### Immunodetection

#### Sample preparation

Cells were collected by centrifugation and resuspended in lysis buffer (5 mmol L^-1^ EDTA, 0.5 mol L^-1^ NaCl, 8% glycerol, 1 μg/mL pepstatin A, 1 mmol L^-1^ PMSF, 25 mmol L^-1^ phosphate buffer, pH 8.0) corresponding to a suspension of 700 μL (OD_600_ 30). For cell disruption, this suspension was combined with 500 μL of glass beads (0.5 mm, Sartorius, Germany) and treated seven times in a Thermo Savant Fastprep FP120 homogenizer (speed 6.00 m/s for 30 s; cooling interval of 30 s between treatments).

#### Western blotting and immunostaining

Proteins were separated on 12% SDS-PAGE gels prior to electroblotting onto PVDF membranes (Bio-Rad, Hercules, USA) at 12 volts for 45 min. On each lane, the same sample volume was loaded corresponding to an identical OD_600_. The membranes were blocked with 5% skimmed milk (Difco, France) in PBS containing 0.5% Tween 20 (PBS-T) for 2 h. After washing the membranes with PBS-T, the mouse anti-HDEL antibody (2E7) (sc-53472; 1:1000 dilution, Santa Cruz Biotechnology, USA) was added and the membranes incubated for 1 h at room temperature. After washing with PBS-T, the secondary anti-mouse antibody (1:5000 dilution, Calbiochem, Germany) was added and incubation continued for 1 h. Immunostaining was done using 3,3’,5,5’ tetramethylbenzidine (Sigma, Germany) as substrate. The mouse anti-HDEL antibody (2E7) binds only to the six *P. pastoris* proteins containing the C-terminal HDEL sequence, namely KRE5 (166.2 kDa), SEC12 (116.2 kDa), LHS1 (99.5 kDa), KAR2 (74.2 kDa), PDI1 (57.8 kDa), and MPD1(33.5 kDa) [33].

## Competing interests

The authors declare that they have no competing interests.

## Authors’ contributions

AV did the experimental work, analyzed the data and prepared a first draft of the manuscript. MN contributed to protein identification by Maldi-ToF. UR conceived and directed the study and prepared the final manuscript. All authors read and approved the final manuscript.

## Supplementary Material

Additional file 1: Table S1 All identified intracellular proteins of *P. pastoris* X-33 classified into functional categories. **Figure S1.** Representative 2D gels of the intracellular proteome (A) before and (B) during IP production. **Table S2.** Change of the intracellular proteome in response to IP production during methanol induction. **Figure S2.** Change of the intracellular proteome of *P. pastoris* X-33 in response to secretory insulin precursor production with special attention to ERAD and UPR related proteins (proteome analysis of replicate cultivation). Click here for file
