# Supplementary material for: Decrease of UPR- and ERAD-related proteins in Pichia pastoris during methanol-induced secretory insulin precursor production in controlled fed-batch cultures
Source: Microb Cell Fact. 2014 Feb 13;13:23. doi: 10.1186/1475-2859-13-23 (PMC3930904; doi:10.1186/1475-2859-13-23)

Additional file (Vanz et al 2014)

Table S1 - All identified intracellular proteins of *P. pastoris* X-33 classified into functional categories

NCBI Accession-No[1]	Locus ID[1]	Gene name[2]	Protein name[2]	MW (KDa)[3]	p*I*[3]	Mascot Score	Spot No[4]
Functional category[1]							
**1. Metabolism**							
**1.1 Carbohydrate metabolism**							
**Methanol metabolism**							
XP_002494271	PAS_chr4_0821	*AOX1*	Alcohol oxidase	74.5	6.0	124	14*,15*,148*, 156* (74.5 kDa), 163* (~25 kDa)
XP_002492075	PAS_chr2-2_0131	*CTA1*	Catalase A	58.1	6.6	183	18*
XP_002493065	PAS_chr3_0832	*DAS1*	Transketolase (Dihydroxyacetone synthase), similar to Tkl2p	79.1	6.0	174	153*, 223*,16*
XP_002493071	PAS_chr3_0841	*DAK*	Dihydroxyacetone kinase	65.4	5.4	114	57*, 58*
XP_002493270	PAS_chr3_1028	*FLD1*	S-(hydroxymethyl) glutathione dehydrogenase	41.4	6.1	81[5]	24*, 25*, 28*
XP_002493100	PAS_chr3_0867	*FGH1*	Non-essential intracellular esterase that can function as an S-formylglutathione hydrolase	33.4	6.3	131	49*, 54*
XP_002493171	PAS_chr3_0932	*FDH1*	NAD(+)-dependent formate dehydrogenase	40.4	6.6	119	29*, 30*, 31*, 32*, 33* (40 kDa), 61* (~10 kDa), 80*, 81* (~17 kDa), 87*, 89* (~30 kDa)
**Glycolysis**							
XP_002494228	PAS_chr4_0783	*GUT1*	Glycerol kinase	68.8	5.3	104	145*

NCBI Accession-No[1]	Locus ID[1]	Gene name[2]	Protein name[2]	MW (KDa)[3]	p*I*[3]	Mascot Score	Spot No[4]
**Functional category[1]**							
XP_002493103	PAS_chr3_0868	*FBP1*	Fructose-1,6-bisphosphatase	38.4	6.1	118	41*
XP_002491345	PAS_chr2-1_0437	*TDH1*	Glyceraldehyde-3-phosphate dehydrogenase, isozyme 3	35.7	6.2	106	45*, 48*, 95*,118*
XP_002489713	PAS_chr1-1_0072	*FBA1*	Fructose 1,6-bisphosphate aldolase	39.9	6.0	186	115*
XP_002490411	PAS_chr1-4_0292	*PGK1*	3-Phosphoglycerate kinase	44.1	7.8	152	210*, 211*
XP_002493191	PAS_chr3_0951	*TPI1*	Triose phosphate isomerase	27.1	5.7	244	112*
XP_002493059	PAS_chr3_0826	*GMP1*	Tetrameric phosphoglycerate mutase	28.0	6.0	165	65*
XP_002492293	PAS_chr3_0082	*ENO2*	Enolase I	46.5	5.4	102	22*, 59*, 150*
**Ethanol metabolism**							
CAY67035	PAS_c034_0018	*ADH*	Alcohol dehydrogenase	38.1	6.3	195	78*
XP_002491382	PAS_chr2-1_0472	*ADH3*	Mitochondrial alcohol dehydrogenase isozyme III	37.3	5.8	145	42*, 44*, 55*, 93*, 96*
**Citrate cycle (TCA cycle)**							
XP_002489764	PAS_chr1-1_0475	*CIT1*	Citrate synthase	51.9	7.8	102	27*
XP_002489444	PAS_chr1-3_0104	*ACO1*	Aconitase	85.0	5.7	77	129*
XP_002491128	PAS_chr2-1_0238	*MDH1*	Mitochondrial malate dehydrogenase	36.5	5.3	129	43*, 47*, 53*, 56*, 103*, 141*
**Pentose phosphate pathway**							
XP_002492495	PAS_chr3_0277	*6PGD [GND]*	6-Phosphogluconate dehydrogenase (decarboxylating)	54.2	5.9	116	23*
XP_002491849	PAS_chr2-2_0337	*TAL1*	Transaldolase	35.7	5.1	111	109*
**Sugar metabolism and others**							
XP_002490557	PAS_chr1-4_0426	*BGL2*	Endo-beta-1,3-glucanase	34.2	4.1	109	158*
XP_002493105	PAS_chr3_0870	*VIG9 [PSA1]*	GDP-mannose pyrophosphorylase (mannose-1-phosphate guanyltransferase)	40.1	6.0	95	215*

NCBI Accession-No[1]	Locus ID[1]	Gene name[2]	Protein name[2]	MW (KDa)[3]	pI[3]	Mascot Score	Spot No[4]
Functional category[1]							
**Pyruvate metabolism**							
XP_002492397	PAS_chr3_0188	*PDC1*	Major of three pyruvate decarboxylase isozymes	61.5	5.6	118	11*, 12*
XP_002491703	PAS_chr2-1_0769	*PYK2*	Pyruvate kinase	55.9	6.1	190	161*
**1.2 Energy Metabolism**							
**Oxidative phosphorylation**							
XP_002493409	PAS_c131_0021	*[VMA2]*	Vacuolar ATP synthase subunit B	55.4	5.3	159	167*
XP_002492803	PAS_chr3_0576	*atpA*	Alpha subunit of the F1 sector of mitochondrial F1F0 ATP synthase	59.0	9.1	76	52*, 66*, 125*
XP_002492039	PAS_chr2-2_0165	*atpD*	Beta subunit of the F1 sector of mitochondrial F1F0 ATP synthase	54.0	5.2	83	6*, 35*, 68*
XP_002494179	PAS_chr4_0737	*MCR1*	Mitochondrial NADH-cytochrome b5 reductase	33.6	8.8	151	67*
**1.3 Nucleotide metabolism**							
XP_002492154	PAS_chr2-2_0059	*YNK1*	Nucleoside diphosphate kinase	17.0	6.2	83	121*
**1.4 Amino acid metabolism**							
XP_002490090	PAS_chr1-1_0432	*ILV5*	Acetohydroxyacid reductoisomerase	44.4	7.7	92	37*
XP_002491046	PAS_chr2-1_0160	*metE [MET6]*	Cobalamin-independent methionine synthase	85.9	5.9	193	107*, 128*
XP_002492638	PAS_chr3_0410	*CAR2*	L-Ornithine transaminase	47.5	5.9	249	200*
XP_002490747	PAS_chr1-4_0608	*ARO8*	Aromatic aminotransferase I	54.2	5.4	172	165*
XP_002492166	PAS_chr2-2_0048		Dihydrolipoamide dehydrogenase	52.6	6.3	113	13*
XP_002493126	PAS_chr3_0890	*SAHH*	S-adenosyl-L-homocysteine hydrolase	49.2	5.4	147	151*
XP_002493112	PAS_chr3_0876	*SAM2*	S-adenosylmethionine synthetase	42.6	6.1	96	205*

NCBI Accession-No[1]	Locus ID[1]	Gene name[2]	Protein name[2]	MW (KDa)[3]	p*I*[3]	Mascot Score	Spot No[4]
**Functional category[1]**							
**1.5 Metabolism of cofactors and vitamins**							
XP_002493470	PAS_chr4_0065	*THI5*	Protein involved in synthesis of the thiamine precursor hydroxymethylpyrimidine (HMP)	38.7	6.3	94	46*
XP_002493989	PAS_chr4_0550	-	Pyridoxine 4-dehydrogenase	36.3	5.6	97	138*
XP_002492875	PAS_chr3_0648	*THI4*	Thiazole synthase	37.3	6.2	92	113*
**2. Genetic information processing**							
**2.1 Transcription and translation**							
XP_002491688	PAS_chr2-1_0755	*EGD2*	Alpha subunit of the heteromeric nascent polypeptide-associated complex (NAC)	21.6	4.6	78[5]	73*
XP_002489404	PAS_chr1-3_0068	*RPP0*	Conserved ribosomal protein P0 similar to rat P0, human P0, and E. coli L10e	33.7	4.6	128	72*
XP_002491277	PAS_chr2-1_0376	*EFB1*	Putative GTPase, member of the Obg family	23.2	4.3	122	108*
XP_002490107	PAS_chr1-1_0449	*PST2*	Protein with similarity to members of a family of flavodoxin-like proteins	20.9	6.4	73	86*
XP_002493447	PAS_chr4_0041	-	Protein component of the large (60S) ribosomal subunit	15.1	5.8	86	94*
XP_002489653	PAS_chr1-1_0019	*SUB2*	Component of the TREX complex required for nuclear mRNA export	49.6	5.4	91	166*
XP_002490604	PAS_chr1-4_0471	*RPS0*	Protein component of the small (40S) ribosomal subunit, nearly identical to Rps0Bp	29.3	4.6	72	71*
XP_002493313	PAS_chr3_1071	*CAM1*	Translation elongation factor EF-1 gamma	24.3	6.4	104	78*, 137*
XP_002493749	PAS_chr4_0327	*GCN1*	Positive regulator of the Gcn2p kinase activity, forms a complex with Gcn20p	30.4	5.6	79	101*
**2.2 Sorting and degradation**							
XP_002492588	PAS_chr3_0365	*SSC1*	Mitochondrial matrix ATPase	69.7	5.4	152	106*
XP_002490239	PAS_chr1-4_0130	*HSP90*	Heat shock protein Hsp90	80.9	4.8	81	1*, 2*
XP_002489443	PAS_chr1-3_0102	*clpB [HSP104]*	Heat shock protein that cooperates with Ydj1p (Hsp40) and Ssa1p (Hsp70)	100.6	5.3	140	315*

NCBI Accession-No[1]	Locus ID[1]	Gene name[2]	Protein name[2]	MW (KDa)[3]	p*I*[3]	Mascot Score	Spot No[4]
**Functional category[1]**							
XP002494360.1	PAS_FragD_0026	*[CDC48]*	ATPase in ER, nuclear membrane and cytosol with homology to mammalian p97	91.4	4.8	108	146*
CCA38590	PP7435Chr2-0908	*AAP1*	Aminopeptidase N	23.0	4.7	142	316*
XP_002493087	PAS_chr3_0856	*UBA1*	Ubiquitin activating enzyme (E1)	115.6	5.0	142	325*
**2.3 Folding catalysts**							
CAC33587	PAS_chr4_0844	*PDI*	Protein disulfide isomerase	58.1	4.6	161	134*
XP_002493562	PAS_chr4_0158	*CPNA [HSP60]*	Tetradecameric mitochondrial chaperonin	60.5	5.1	115	4*
XP_002489608	PAS_chr1-3_0264	*[CPR1]*	Cytoplasmic peptidyl-prolyl cis-trans isomerase (cyclophilin)	18.1	6.1	99	92*
XP_002493991	PAS_chr4_0552	*HSP70-HSA1 [SSA1]*	ATPase involved in protein folding and nuclear localization signal (NLS)-directed	69.7	4.1	89	207*
XP_002492959	PAS_chr3_0731	*HSP70 [SSB]*	Cytoplasmic ATPase that is a ribosome-associated molecular chaperone	66.6	5.1	100	209*
XP_002491027	PAS_chr2-1_0140	*KAR2*	ATPase involved in protein import into the ER	74.2	4.8	146	133*
XP_002492443	PAS_chr3_0230	*HSP70 [SSA3]*	ATPase involved in protein folding and the response to stress	70.9	5.1	102	76* (70.9 KDa)<br>105* (~25 KDa)
**3. Cellular processes**							
**3.1 Transport and catabolism**							
XP_002490181	PAS_chr1-4_0071	*SOD2*	Mitochondrial superoxide dismutase	25.2	7.9	85	216*
XP_002493819	PAS_chr4_0391	*[TOM40]*	Component of the TOM (translocase of outer membrane) complex	42.8	5.6	78	36*
XP_002492446	PAS_chr3_1169	*ACT1*	Actin	41.9	5.3	148	21*
**3.2 Cell growth and death**							
XP_002490987	PAS_chr2-1_0809	*BMH1*	14-3-3 protein homolog	29.1	4.8	85	75*
XP_002492031	PAS_chr2-2_0172	*NAP1*	Protein that interacts with mitotic cyclin Clb2p	49.0	4.3	115	135*

NCBI Accession-No[1]	Locus ID[1]	Gene name[2]	Protein name[2]	MW (KDa)[3]	p*I*[3]	Mascot Score	Spot No[4]
Functional category[1]							
**4. Other**							
**4.1 Stress response**							
XP_002491977	PAS_chr2-2_0220	*TSA1*	Thioredoxin peroxidase	21.5	4.9	125	77*
XP_002490091	PAS_chr1-1_0433	*PRX1*	Mitochondrial peroxiredoxin (1-Cys Prx) with thioredoxin peroxidase activity	25.1	5.7	142	82*, 111*
BAH80186	PAS_chr2-2_0480	*TRR1*	Thioredoxin reductase 1	35.3	5.5	93	225*
XP_002491413	PAS_chr2-1_0502	-	Thiol-specific peroxiredoxin	19.3	8.6	106	120*
**5. Unclassified**							
XP_002491418	PAS_chr2-1_0853	-	Hypothetical protein	57.0	6.0	84	7*, 8*, 91*
XP_002489760	PAS_chr1-1_0118	-	Laminin subunit gamma-1	46.3	7.0	73	142*, 157*
XP_002491208	PAS_chr2-1_0313	-	Bifunctional enzyme with alcohol dehydrogenase and glutathione-dependent formaldehyde dehydrogenase	39.1	5.8	92	38*, 39*, 40*
XP_002490174	PAS_chr1-4_0063	-	G-protein beta subunit and guanine nucleotide dissociation inhibitor for Gpa2p	35.0	6.3	104	116*
XM_002490789	PAS_FragB_0024	-	Inositol 2-dehydrogenase	38.0	5.7	79	139*

1 Accession numbers and locus IDs are according to the NCBI Reference Sequence database (http://www.ncbi.nlm.nih.gov/RefSeq/). Functional classifications are mostly according to KEGG PATHWAY database (http://www.genome.jp/kegg/metabolism.html). The data are according to databases on 01.10.12.

2 Gene/protein names are according to the sequenced genome of *P. pastoris* GS115 (http://www.uniprot.org/). If no name was found for *P. pastoris* GS115, a Blast search of the respective *P. pastoris* GS115 gene/protein against different *P. pastoris* strains was carried out in the following order: search against ATCC 76273 / CBS 7435 / CECT 11047 / NRRL Y-11430 / Wegner 21-1 and *P. pastoris* (yeast). Gene/protein names from other *P. pastoris* strains were only adopted in case of 100% sequence identity (http://www.uniprot.org/). Gene names in brackets are from *S. cerevisiae* if the names differ for both yeasts (http://www.uniprot.org/). The data are according to database on 01.10.12.

3 Theoretical molecular mass (MM) and isoelectric point (p*I*) not considering potential posttranslational modifications.

4 "Spot-No" indicates the serial number of all identified protein spots of the intracellular proteome of *P. pastoris* X-33. Proteins appearing in multiple spots indicate the existence of isoforms which probably derive from posttranslational modification events such as phosphorylation, glycosylation or limited proteolysis. Two representative 2D gels of the intracellular proteome from cell samples taken at the end of glycerol phase and after 48 hours of growth on methanol with the respective spot numbers are shown in Figure S1 A and B. Spot numbers in this table and the Figure S1 A and B are marked by an asterix (*) for simplified searchability. Fragments of full-length proteins, AOX1, FDH1 and SSA3, were identified and the respective Spot No and MW are indicated.

5 Score obtained from MS/MS analysis.

Figure S1 A and B - Representative 2D gels of the intracellular proteome before and during IP production

The samples of *P. pastoris X-33* were taken at the end of the glycerol phase (A) and after 48 h of growth on methanol (B). Spot numbers correspond to numbers in the Table S1 and are marked by an asterix (*) for simplified searchability.

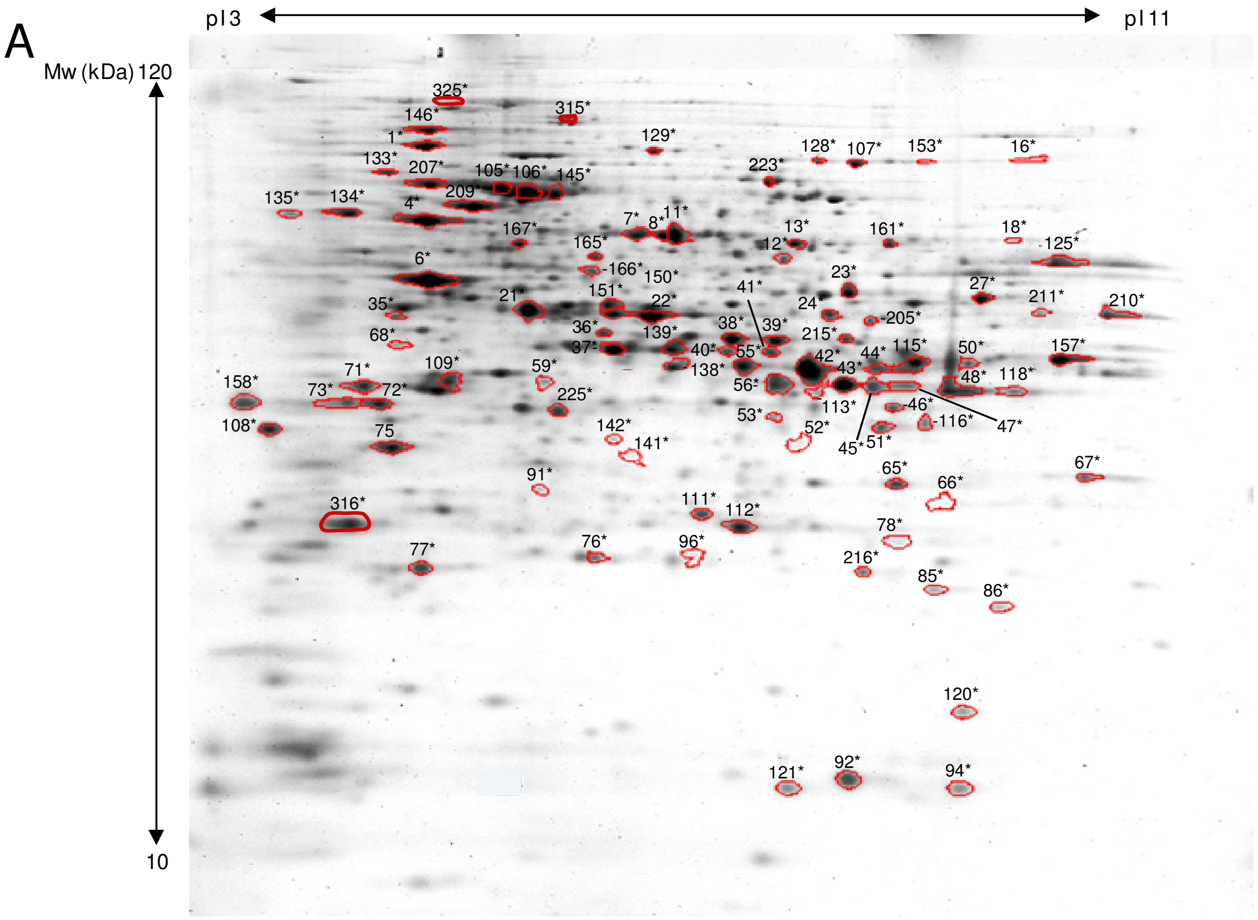

B

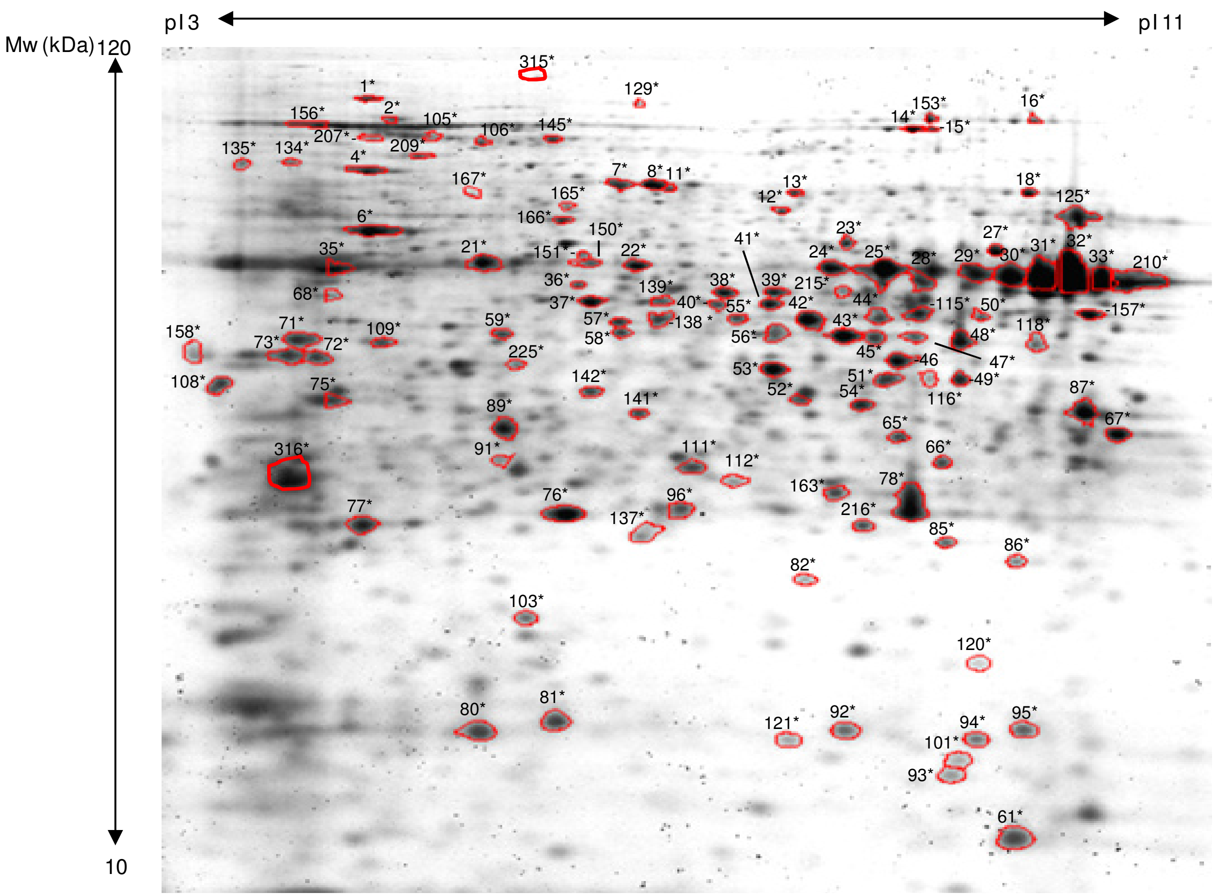
pI 3
pI 11
Mw (kDa)
120
10
315*
1*
129*
2*
156*
153*
16*
14*
15*
105*
207*
106*
145*
209*
135*
134*
4*
7*
8*
11*
167*
13*
18*
165*
12*
125*
166*
6*
23*
150*
27*
41*
35*
21*
151*
22*
24*
25*
31*
32*
28*
29*
30*
33*
210*
36*
38*
39*
215*
139*
68*
37*
40*
55*
42*
44*
115*
50*
157*
57*
138*
43*
118*
71*
109*
59*
158*
73*
72*
58*
56*
48*
225*
45*
46
47*
53*
51*
49*
108*
142*
75*
52*
54*
116*
87*
141*
89*
65*
67*
316*
91*
111*
66*
112*
163*
78*
76*
96*
77*
137*
216*
85*
86*
82*
103*
120*
80*
81*
92*
95*
121*
94*
101*
93*
61*

Table S2 - Change of the intracellular proteome in response to IP production during methanol induction

Samples before methanol addition at the end of the glycerol batch phase and 6, 18, 48 and 120 hours after the onset of methanol feeding were analyzed and the protein changes are shown (numbers. log2 changes; n.a. not analyzable, color code: light orange to red, increase, light green to dark green: decrease, yellow: no significant change).

Functional category	Gene name[1]	Gene ID	0h[2]	6h	18h	48h	120h
**Carbohydrate metabolism**							
Methanol metabolism	*AOX1*	PAS_chr4_0821	0.0	7.4	9.4	9.2	9.5
	AOX1 (~25 kDa)[3]		0.0	7.1	7.1	8.1	8.1
	CTA1	PAS_chr2-2_0131	0.0	1.7	2.0	1.5	2.3
	DAS1	PAS_chr3_0832	0.0	0.1	0.9	-0.1	-0.2
	DAK	PAS_chr3_0841	0.0	n.a	6.5	8.1	8.7
	FLD1	PAS_chr3_1028	0.0	9.4	9.9	10.7	10.8
	FGH1	PAS_chr3_0867	0.0	7.0	8.4	8.8	9.0
	FDH1	PAS_chr3_0932	0.0	10.7	11.6	12.3	12.3
	FDH1 (~30 kDa)[3]		0.0	6.2	8.3	10.1	10.5
	FDH1 (~17 kDa)[3]		0.0	0.0	8.1	9.6	10.5
	FDH1 (~10 kDa)[3]		0.0	0.0	7.1	9.3	9.4
Glycolysis	*GUT1*	PAS_chr4_0783	0.0	-0.4	0.1	-1.6	n.a
	FBP1	PAS_chr3_0868	0.0	0.1	0.6	0.6	0.6
	TDH1	PAS_chr2-1_0437	0.0	0.6	-0.3	-0.6	-1.1
	FBA1	PAS_chr1-1_0072	0.0	0.2	-0.6	-1.1	-1.0
	PGK1	PAS_chr1-4_0292	0.0	-1.0	n.a	1.1	0.4
	TPI1	PAS_chr3_0951	0.0	0.1	-0.4	-2.1	-2.2
	GMP1	PAS_chr3_0826	0.0	0.0	-0.2	-0.8	-1.1
	ENO2	PAS_chr3_0082	0.0	0.1	0.0	-1.1	-0.8
Ethanol metabolism	*ADH3*	PAS_chr2-1_0472	0.0	0.0	0.0	-0.7	-0.6

Functional category	Gene name[1]	Gene ID	0h[2]	6h	18h	48h	120h
Citrate cycle (TCA)	*CIT1*	PAS_chr1-1_0475	0.0	1.0	0.7	0.3	0.4
	ACO1	PAS_chr1-3_0104	0.0	0.5	0.1	-1.0	-0.4
	MDH1	PAS_chr2-1_0238	0.0	0.1	-0.2	-0.1	0.5
Pentose phosphate pathway	*6PGD [GND]*	PAS_chr3_0277	0.0	0.9	0.3	-0.5	-0.9
	TAL1	PAS_chr2-2_0337	0.0	1.1	0.4	-1.0	-0.4
Sugar metabolism	*BGL2*	PAS_chr1-4_0426	0.0	0.2	-0.1	-1.0	-0.9
	VIG9 [PSA1]	PAS_chr3_0870	0.0	0.4	-0.9	-0.9	n.a
Pyruvate metabolism	*PDC1*	PAS_chr3_0188	0.0	0.0	-0.5	-1.4	-2.0
	PYK2	PAS_chr2-1_0769	0.0	0.6	-0.5	-2.3	-1.9
**Energy metabolism**							
Oxidative phosphorylation	*[VMA2]*	PAS_c131_0021	0.0	-0.1	-1.0	-1.2	-1.6
	atpA	PAS_chr3_0576	0.0	0.8	0.3	0.4	1.1
	atpD	PAS_chr2-2_0165	0.0	-0.2	0.4	-0.7	0.1
	MCR1	PAS_chr4_0737	0.0	0.2	0.9	0.2	1.2
**Nucleotide metabolism**							
	YNK1	PAS_chr2-2_0059	0.0	-0.2	-0.6	-1.0	-1.4
**Amino acid metabolism**							
	ILV5	PAS_chr1-1_0432	0.0	0.2	-0.1	-0.7	-0.1
	metE [MET6]	PAS_chr2-1_0160	0.0	0.8	-0.7	n.a	n.a
	CAR2	PAS_chr3_0410	0.0	0.1	-0.3	n.a	-0.3
	ARO8	PAS_chr1-4_0608	0.0	1.3	0.6	-0.1	0.5
	-	PAS_chr2-2_0048	0.0	0.6	-0.4	-0.7	-0.2
	SAHH	PAS_chr3_0890	0.0	0.0	-0.5	-1.6	-0.4
	SAM2	PAS_chr3_0876	0.0	0.9	-0.3	0.4	-0.1
**Metabolism of cofactors and vitamins**							
	-	PAS_chr4_0550	0.0	0.0	-0.7	-0.6	-1.2
	THI5	PAS_chr4_0065	0.0	1.5	2.3	1.0	-0.8
	THI4	PAS_chr3_0648	0.0	1.9	2.3	0.5	0.1

Functional category	Gene name[1]	Gene ID	0h[2]	6h	18h	48h	120h
**Genetic information processing**							
Transcription and translation	*EGD2*	PAS_chr2-1_0755	0.0	-0.3	-0.9	-0.3	-0.2
	RPP0	PAS_chr1-3_0068	0.0	0.7	-0.1	-0.4	-0.2
	EFB1	PAS_chr2-1_0376	0.0	-0.3	-0.4	-0.9	-1.0
	PST2	PAS_chr1-1_0449	0.0	0.3	0.3	-0.8	-0.5
	-	PAS_chr4_0041	0.0	0.1	-0.4	-0.1	-1.0
	SUB2	PAS_chr1-1_0019	0.0	0.6	0.0	0.1	1.2
	RPS0	PAS_chr1-4_0471	0.0	-0.3	-0.4	-0.2	-2.5
	CAM1	PAS_chr3_1071	0.0	0.8	2.2	3.5	2.5
	GCN1	PAS_chr4_0327	0.0	n.a	7.4	7.1	6.5
Sorting and degradation	*SSC1*	PAS_chr3_0365	0.0	-0.3	-1.3	-2.6	-1.0
	Hps90	PAS_chr1-4_0130	0.0	-0.6	-1.6	-0.9	n.a
	clpB [HSP104]	PAS_chr1-3_0102	0.0	0.5	-1.2	-2.2	n.a
	[CDC48]	PAS_FragD_0026	0.0	-0.5	-1.4	-3.6	-3.4
	AAP1	PP7435_Chr2-0908	0.0	-0.5	-0.5	-0.8	-0.6
	UBA1	PAS_chr3_0856	0.0	-0.5	-1.5	n.a	n.a
Folding catalysts	*PDI*	PAS_chr4_0844	0.0	-0.2	-1.2	-2.7	-1.7
	CPNA [HSP60]	PAS_chr4_0158	0.0	0.0	-0.5	-1.5	-0.2
	[CPR1]	PAS_chr1-3_0264	0.0	-0.2	-0.7	-1.3	-1.4
	HSP70-HSA1 [SSA1]	PAS_chr4_0552	0.0	-0.7	-1.5	-2.9	-2.9
	HSP70 [SSB]	PAS_chr3_0731	0.0	0.1	-0.8	-2.5	-2.2
	KAR2	PAS_chr2-1_0140	0.0	0.1	-1.5	n.a	-1.4
	HSP70 [SSA3]	PAS_chr3_0230	0.0	-0.4	-1.0	-2.7	-2.2
	HSP70 [SSA3] (~ 25 KDa)[3]		0.0	0.3	1.4	1.8	2.4
**Cellular processes**							
Transport and catabolism	*SOD2*	PAS_chr1-4_0071	0.0	0.3	0.0	-0.1	0.2
	[TOM]	PAS_chr4_0391	0.0	0.4	-0.1	-1.0	1.1
	ACT1	PAS_chr3_1169	0.0	0.1	0.2	0.0	-0.2
Cell growth and death	*BMH1*	PAS_chr2-1_0809	0.0	-0.1	0.0	-0.8	-0.9
	NAP1	PAS_chr2-2_0172	0.0	-0.7	-0.5	-0.4	-1.4

Functional category	Gene name[1]	Gene ID	0h[2]	6h	18h	48h	120h
**Others**							
Stress response	*TSA1*	PAS_chr2-2_0220	0.0	-0.2	0.4	-0.1	0.6
	PRX1	PAS_chr1-1_0433	0.0	0.0	0.3	-0.1	0.4
	TRR1	PAS_chr2-2_0480	0.0	0.3	-0.1	-1.8	-1.2
	-	PAS_chr2-1_0502	0.0	0.3	-0.4	-1.4	-1.3
Unclassified	-	PAS_chr2-1_0853	0.0	0.5	0.1	-0.1	0.6
	-	PAS_chr1-1_0118	0.0	0.5	-0.1	-0.7	-0.4
	-	PAS_chr2-1_0313	0.0	-0.1	-0.3	-0.7	-0.3
	-	PAS_chr1-4_0063	0.0	1.3	0.6	0.1	-0.4
	-	PAS_FragB_0024	0.0	0.2	-0.4	-1.4	-1.2

1 Gene names in brackets are from *S. cerevisiae* if the names differ for both yeasts (http://www.uniprot.org/).

2 When no protein spot was detectable at the end of the glycerol batch phase (methanol metabolism), a virtual number of 0.001 was taken for the spot intensity to allow the calculation of log2 values (spot intensities were normalized using the log2 ratio of induced samples *versus* uninduced samples).

3 Fragments of full-length proteins, AOX1, FDH1 and SSA3, were identified and the respective log2 fold changes are given

Figure S2 A and B - Changes of the intracellular proteome of *P. pastoris* X-33 in response to secretory insulin precursor with special attention to ERAD and UPR related proteins (proteome analysis of replicate cultivation)

(A) Sections of 2D gels representing parts of the intracellular proteome and containing most of ERAD and UPR related proteins at 0, 5, 16, and 72 h after induction with methanol. (B) Representative graphs of the normalized relative abundance changes of each protein spot shown in A. The spot related to fragment SSA3 ~25 kDa is shown in the small insert. Deviating from the initial feeding protocol for IP production, the methanol concentration in this cultivation was not raised stepwise but directly to 2 g L^{-1}. The small map indicates the position of the 2D sections in the entire 2D gel.

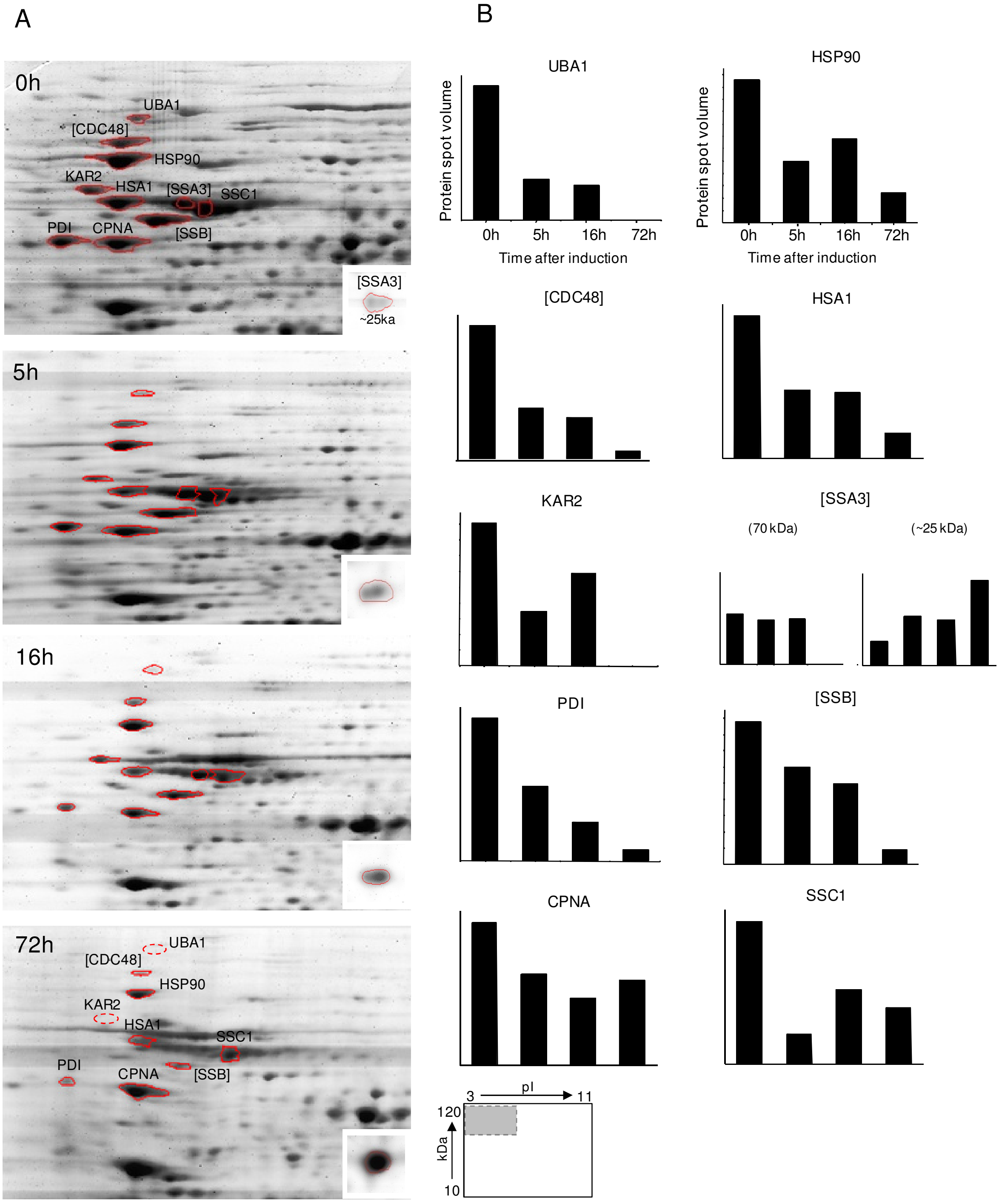

Supplement: Additional file 1: Table S1 — All identified intracellular proteins of P. pastoris X-33 classified into functional categories. Figure S1. Representative 2D gels of the intracellular proteome (A) before and (B) during IP production. Table S2. Change of the intracellular proteome in response to IP production during methanol induction. Figure S2. Change of the intracellular proteome of P. pastoris X-33 in response to secretory insulin precursor production with special attention to ERAD and UPR related proteins (proteome analysis of replicate cultivation). [file 1475-2859-13-23-S1.pdf]
